# Supplementary figures and images for: Genome-wide single-molecule analysis of long-read DNA methylation reveals heterogeneous patterns at heterochromatin that reflect nucleosome organisation
Source: PLoS Genet. 2023 Oct 2;19(10):e1010958. doi: 10.1371/journal.pgen.1010958 (PMC10569558; doi:10.1371/journal.pgen.1010958)

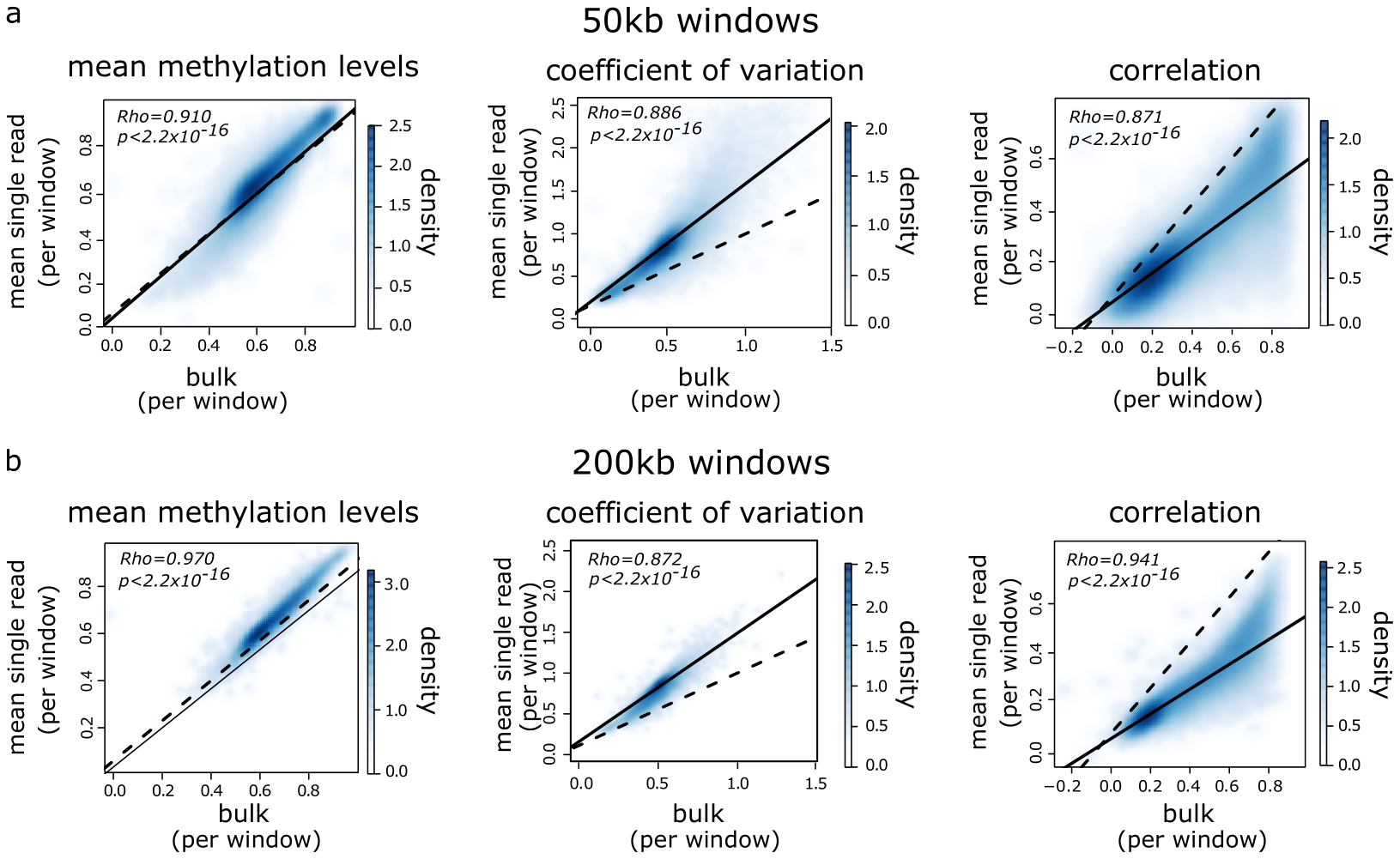

Supplement: S1 Fig — Density scatter plots of mean single-read statistics vs. bulk statistics for 50kb genomic windows (n = 52, 840) (a) and 200kb genomic windows (n = 13, 439) (b). Left: mean methylation level, middle: coefficient of variation, right: correlation between neighbouring CpG sites. Dashed and solid lines show lines of identity and linear models fitted to the data respectively. Spearman correlations are shown, as are p-values from paired t-tests comparing bulk to single-read statistics. (TIF) [file pgen.1010958.s001.tif]

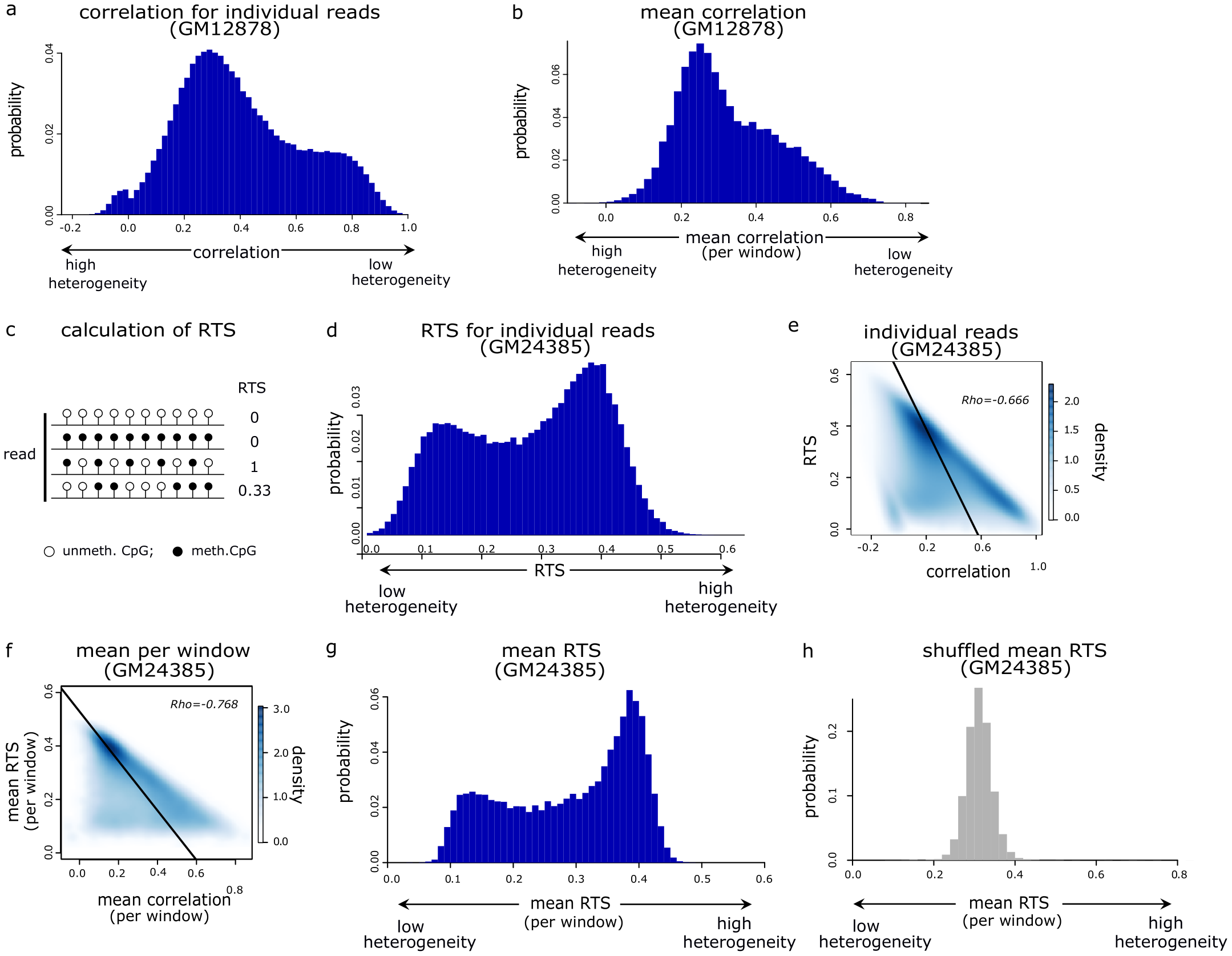

Supplement: S2 Fig — a Histogram showing the correlation distribution for the 1, 331, 213 GM12878 reads containing methylation states for ≥100 CpGs. b Histogram of the mean correlation distribution in GM12878 for 100kb genomic windows (n = 26, 782), calculated using reads that align entirely within each window. c Schematic of RTS values calculated for hypothetical 10 CpG reads. d Histogram showing the RTS distribution for the 2, 908, 181 individual GM24385 reads containing methylation information for ≥ 100 CpGs. e Density scatter plot of the RTS vs. correlation for individual GM24385 reads. f Density scatter plot of the mean RTS vs. mean correlation for 100kb genomic windows in GM24385. In e, f the solid line shows a linear model fitted to the data and the Spearman correlation coefficient is shown in the top right. g Histogram showing the distribution of mean RTS in GM24385 for 100kb windows (n = 26, 951) calculated using reads that align entirely within these windows. h Histogram showing the mean shuffled RTS in GM24385 for 100kb windows (n = 26, 951), calculated using random reads rather than those that align to each window. (TIF) [file pgen.1010958.s002.tif]

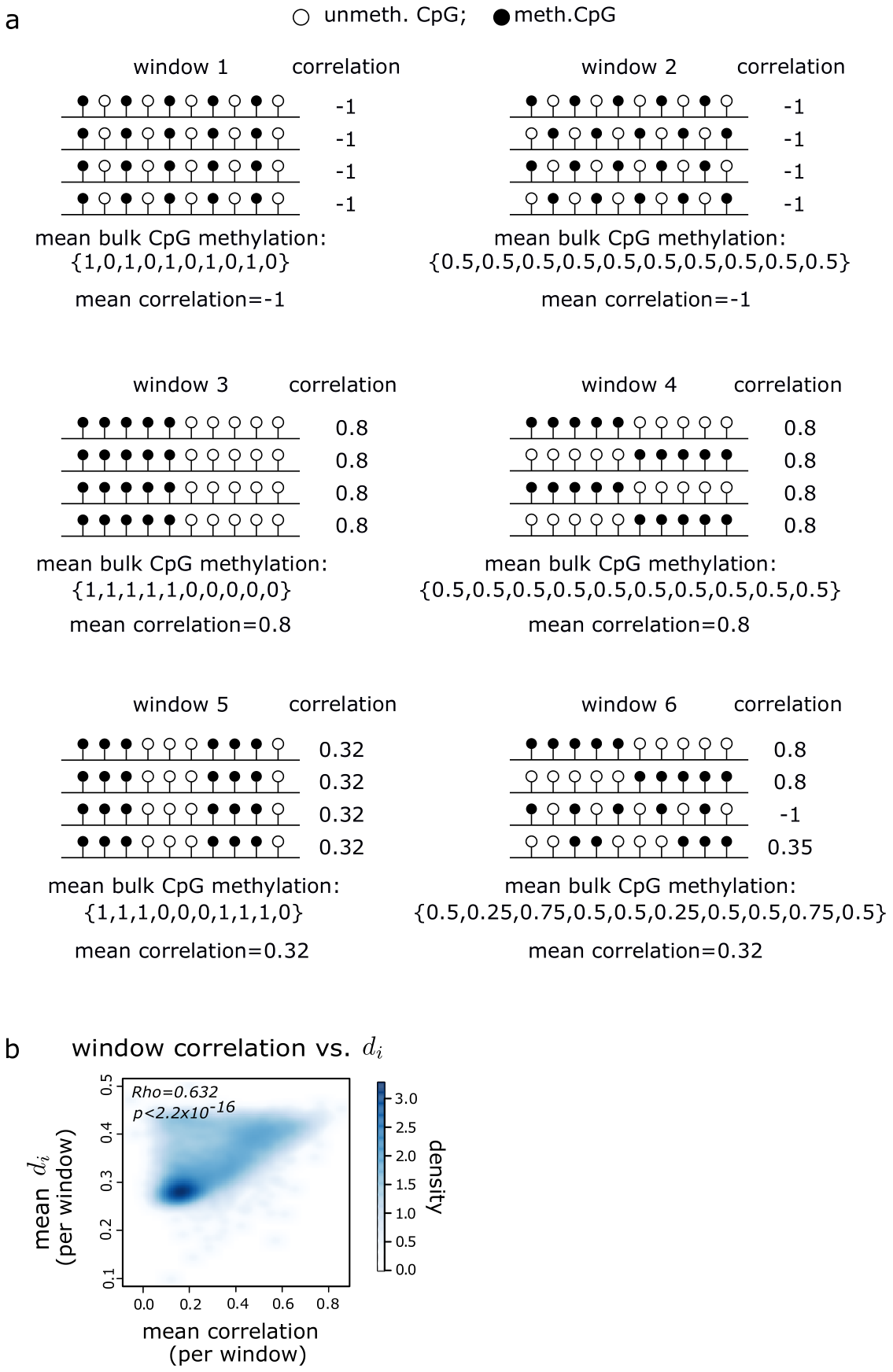

Supplement: S3 Fig — a Shown are six hypothetical examples of genomic windows composed of 10 CpG sites and covered by four reads. For each example, the correlation associated with each read is shown to the right of the read and the mean correlation for the window is shown below each example. The mean bulk methylation of each individual CpG is indicated below the reads. Each pair of examples: windows 1 and 2, windows 3 and 4, windows 5 and 6, show situations where the same mean window correlation is associated with differing degrees of inter-molecular heterogeneity. b Density scatter plot of mean window correlation vs. mean window di for all 100kb genomic windows. Spearman correlation (Rho) is shown along with the corresponding p-value. (TIF) [file pgen.1010958.s003.tif]

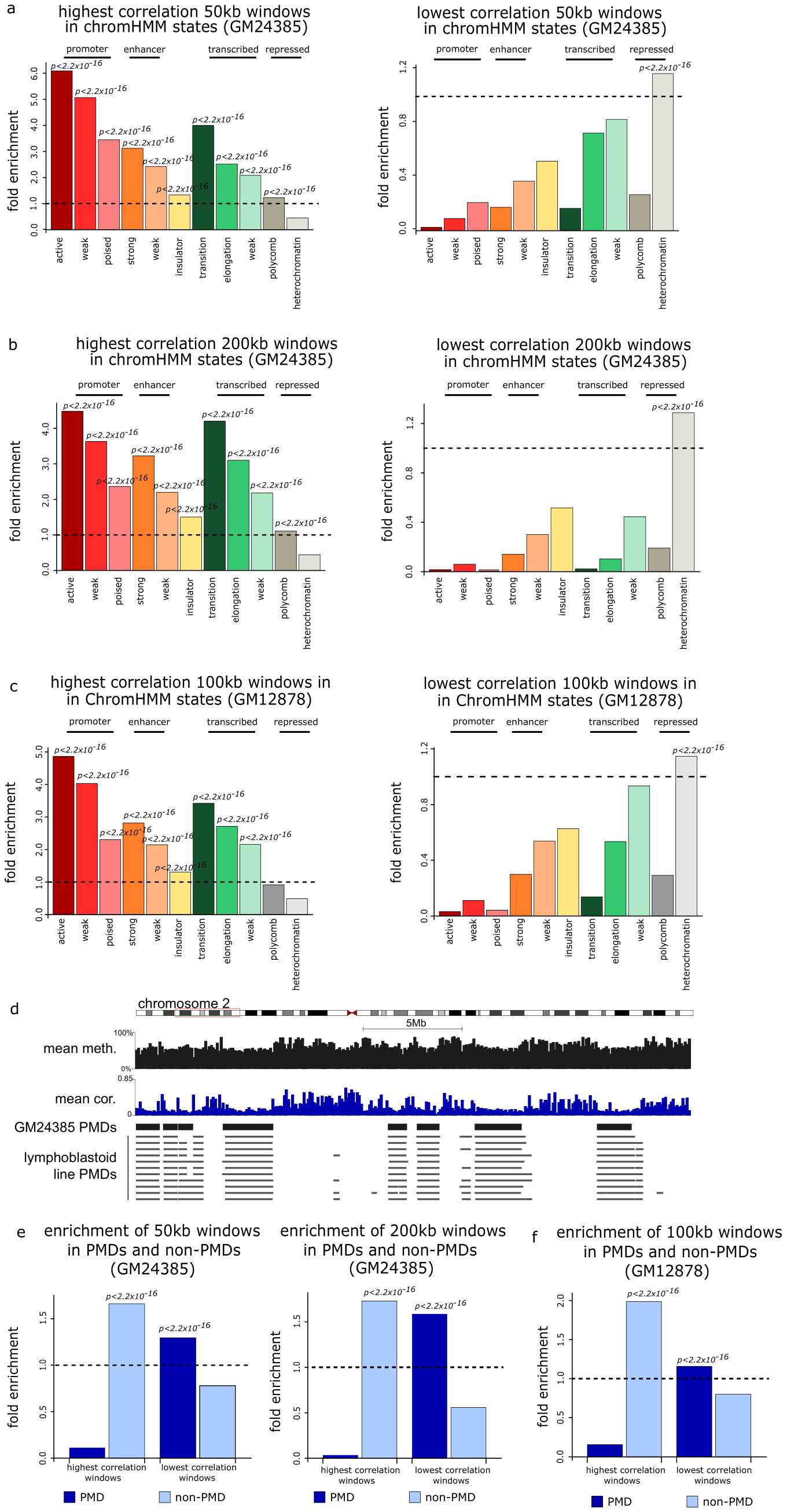

Supplement: S4 Fig — a Barplots showing enrichments of the least and most heterogeneous GM24385 50kb windows in GM12878 chromHMM states. b Barplots showing enrichment of the least and most heterogeneous GM24385 200kb windows in GM12878 chromHMM states. c Barplots showing enrichment of the least and most heterogeneous GM12878 100kb windows in GM12878 chromHMM states. d Genome browser plot showing a representative genomic region with the mean DNA methylation and mean correlation in 100kb windows alongside GM24385 PMD locations and those previously identified in 11 other lymphoblastoid cell lines [26]. e Barplots showing enrichment of the least and most heterogeneous GM24385 50kb windows (left) and 200kb windows (right) in PMDs and non-PMDs. Shown are significant p-values from Wilcoxon tests. f Barplot showing enrichment of the least and most heterogeneous GM12878 100kb windows in PMDs and non-PMDs. Shown are significant p-values from Wilcoxon tests. (TIF) [file pgen.1010958.s004.tif]

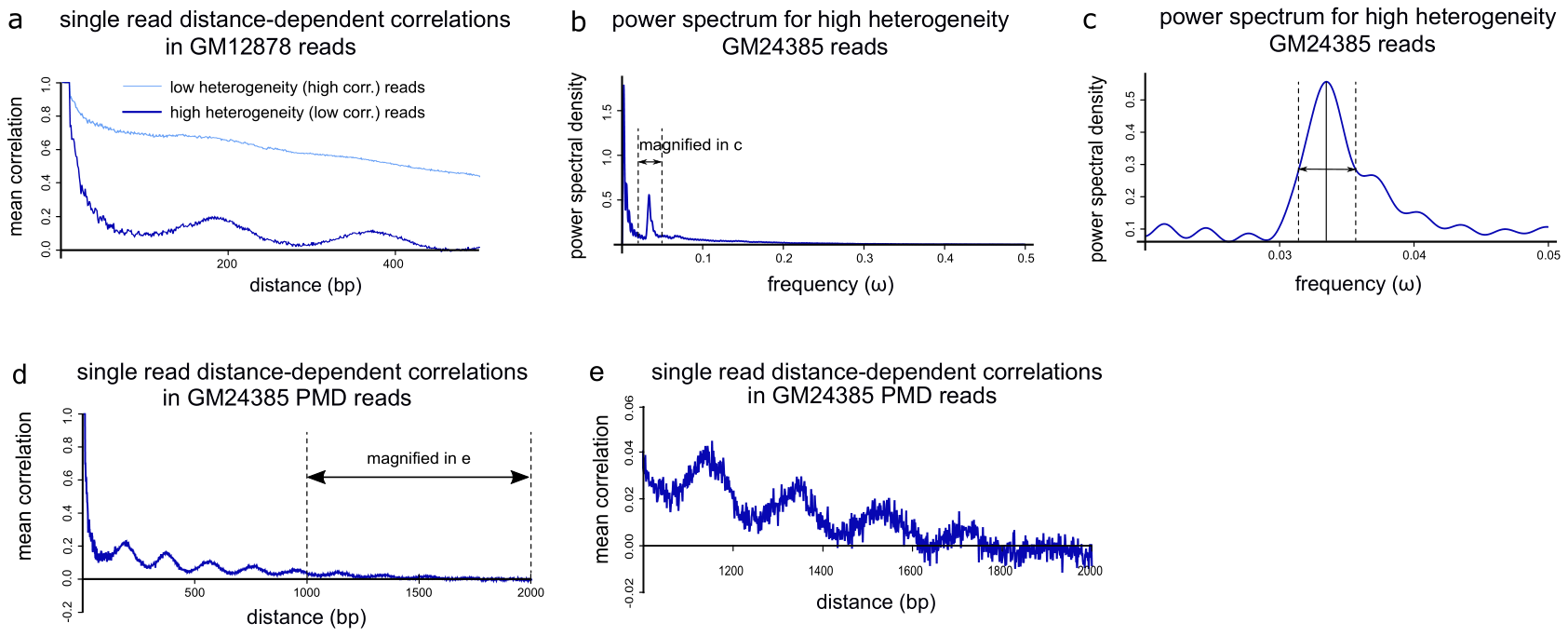

Supplement: S5 Fig — a Plot of mean single-read distance-dependent correlation between CpG sites within 500bp of each other for 714, 789 high heterogeneity GM12878 reads and 407, 536 low heterogeneity GM12878 reads. b Plot of the power spectral density for the mean distance-dependent correlation associated with the high heterogeneity reads. c Magnification of the region indicated in b showing the power spectrum peak at 0.0335 (solid line) and the frequency range for which the power spectrum takes at least half of this maximum value (dotted lines). d Plot of mean single-read distance-dependent correlation between CpG sites within 2kb of each other for 1, 007, 580 GM24385 reads aligning entirely within PMDs. e Magnification of the region indicated in d (i.e. mean single-read distance-dependent correlation for distances 1kb-2kb in GM24385 PMD reads). (TIF) [file pgen.1010958.s005.tif]

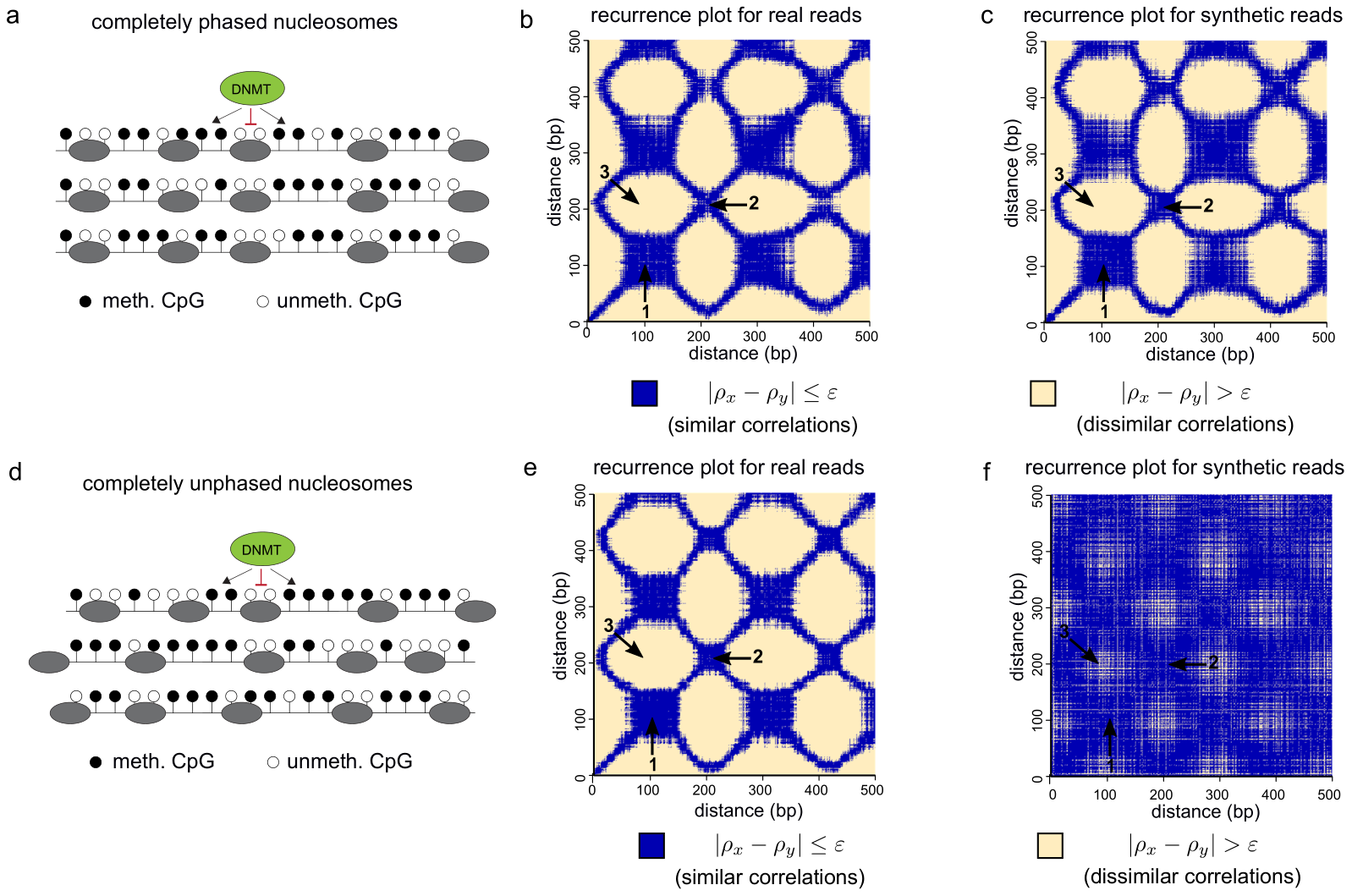

Supplement: S6 Fig — a Schematic of model of the interaction between DNA methylation and nucleosomes in the case of perfect nucleosomal phasing between molecules (see Methods). b,c single-molecule (b) and bulk (c) recurrence plots derived from the perfect phasing model. Here ε = 0.06 for comparison to recurrence plots associated with real data. d Model of completely unphased nucleosomes (Methods). Here it is assumed that nucleosomes are positioned randomly in each molecule. e,f Single-molecule (e) and bulk (f) recurrence plots derived from the completely unphased model. Again ε = 0.06 for comparison to recurrence plots associated with real data. In b,c,e,f, arrows 1 and 2 indicate example regions where the correlations for distances x and y are similar in the single-molecule data generated from the models. Arrow 3 indicates an example region where the correlations for distances x and y are dissimilar in the single-molecule data generated from the models. (TIF) [file pgen.1010958.s006.tif]
