# Supplementary material for: Genome-wide single-molecule analysis of long-read DNA methylation reveals heterogeneous patterns at heterochromatin that reflect nucleosome organisation
Source: PLoS Genet. 2023 Oct 2;19(10):e1010958. doi: 10.1371/journal.pgen.1010958 (PMC10569558; doi:10.1371/journal.pgen.1010958)
Supplement: Attachment — Submitted filename: Kerr_et_al_rebuttal_180823.pdf [file pgen.1010958.s007.pdf]

Response to Reviewers

We thank the reviewers for recognizing the importance of our work and for their supportive and insightful comments that have helped us substantially improve the manuscript. In particular, we provide analyses replicating our results in additional samples and use nanoNOMe-seq data to more directly examine the relationship between DNA methylation and nucleosome occupancy.

We provide a point-by-point response to each comment below. Reviewers' comments are indicated by blue text. Any positions referenced correspond to those in the revised document. We also enclose a revised manuscript, with changes highlighted in red.

Reviewer 1

In this manuscript, Kerr et al. use nanopore long-read DNA sequencing of a human lymphoblastoid cell line to investigate the variability of 5mCG along single molecules (mean length 25 kb). Focusing mainly on the correlation between adjacent CpG sites to measure heterogeneity, the authors extend previous results from short read bisulfite sequencing to show that heterochromatin has more heterogeneous methylation than euchromatin. The long reads enable them to show nucleosomal phasing of methylation over several kb. The methods and results are presented in excellent detail and the analysis is well done. While the biological insights are limited and other long-read papers have touched on single-molecule 5mCG heterogeneity and nucleosome phasing (Lee et al. 2020, Altemose et al. 2022, recently Weigert et al. 2023...), it is to my knowledge the first study to quantify these patterns systematically and genome-wide with long reads.

We appreciate the comments and suggestions made by Reviewer 1 that we feel have improved the manuscript.

Comments:

1. The coefficient of variation does not seem to be a good metric to quantify heterogeneity within a read. For binary data, the mean and number of CpGs already give an excellent account of the heterogeneity (standard deviation is maximum for a mean of 0.5, and null for means of 0 or 1). Using the coefficient of variation unnecessarily weighs in favour of reads with the lowest methylation. For instance, a read with 99 mCGs and 1 CG (1,1,1…,1,0) would have the same standard deviation (0.1) as a read with 1 mCG and 99 mCGs (0,1,1,...,1), and intuitively it makes sense to consider them as being similarly "heterogeneous". However the coefficients of variation would be very different: 0.101 for read1 (considered not very variable in the manuscript), and 10 for read2 (considered very variable in the manuscript). The single read correlation calculation should not be affected by this issue, so the rest of the analysis should be fine.

We appreciate the reviewer's concern that the coefficient of variation is dependent on the mean methylation level within a read. In this analysis, we are comparing a single molecule estimates of variation to that derived from bulk data. We observe that the bulk coefficient of variation is less that the single-molecule coefficient of variation for 99.2% of windows. This indicates that our main point, that heterogeneity is underestimated by bulk measures, holds regardless of methylation level. We have added a comment to the revised manuscript to make it clear that the coefficient of variation is dependent on the mean methylation level, and that this is one reason why the correlation is a more appropriate measure of heterogeneity to use throughout the rest of the paper (starting at line 107):

"We chose to measure single-molecule heterogeneity using the correlation rather than the coefficient of variation since the latter is dependent on the overall mean methylation level and does not take into account information relating to the methylation states of neighbouring CpGs."

2. Using Nanopolish to call methylation may be problematic for two reasons. 1) Nanopolish looks at k-mers (5 or 6 bases) and cannot tell whether adjacent CpG sites have divergent methylation states, it just calls a probability for the k-mer. This might be particularly problematic when looking at short range correlations (lag-1 correlation, and the nucleosome patterning). 2) Nanopolish sometimes has a lot of low confidence calls. Here they would be filtered out, but it's possible this introduces bias against certain motifs/genomic contexts/… Using the latest R9 remora models may circumvent these two issues. At least these potential issues with Nanopolish can be discussed and explored.

We agree that it would be interesting to explore the impact of these issues by using different methylation calling approaches. However, during the revision period we did not have sufficient time to reconstruct our pipeline around alternative methylation callers. As the reviewer suggests, we have now added a paragraph to the discussion describing the potential limitations of Nanopolish as well as the steps we have taken to mitigate their effect (starting at line 545):

"The accuracy of results obtained from the analysis of methylation data is dependent upon the technology used to detect methylation. While we used Nanopolish to call methylation from Nanopore data other methylation callers exist including Guppy, Megalodon, Remora and Dorado from Nanopore, as well as other callers including DeepSignal, Tombo, METEORE and DeepMod [61—64]. Benchmarking studies such as [65,66] have found Nanopolish to be amongst the top-performing methylation callers, both in terms of accuracy and computational efficiency, though such studies have not assayed performance at the single-molecule level. To deal with erroneous methylation calls within single molecules, we filter out low-confidence Nanopolish methylation calls, though we note that such filtering may bias against certain motifs and sequence contexts. We also restrict our attention to single-molecule patterns consisting of a minimum of 100 called CpGs, meaning the impact of a small number of miscalled CpGs when calculating read statistics should be minimal. In addition, Nanopolish is unable to distinguish methylation states between CpGs that lie in very close proximity of each other, leading to the same methylation state being called for CpGs within 10bp of each other. It is possible that this is why we do not observe the 10bp periodicity in the distance-dependent correlation previously observed from bulk bisulfite sequencing data that has been postulated to be representative of DNA winding around the DNA helix [27]. The inability of Nanopolish to distinguish methylation states of CpGs that are very close together could potentially lead to over-estimation of the correlation between neighbouring CpGs in high-density regions of the genome. Nonetheless, an over-estimation in the correlation between neighbouring CpGs would result in an under-estimation of heterogeneity. This means that the high heterogeneity we observe in heterochromatin and PMDs is unlikely be the product of such errors. Improved methylation calling will likely allow additional insight into the nature of single-molecule DNA methylation patterns."

3. Could you apply a Fourier Transform to the autocorrelation in Fig5 to find the period? Is it possible to detect the 10-bp period one would expect from the DNA helix, as has been reported with short reads? Can you estimate the period in different chromatin contexts, to determine whether nucleosome spacing varies between euchromatin and heterochromatin (and finer ChromHMM distinctions)?

In the revised manuscript, we have included an analysis of the power spectrum to calculate the wavelength of the oscillations observed in high heterogeneity reads (starting at line 305):

"In order to determine the wavelength of these oscillations, we examined the power spectrum associated with the distance-dependent correlation up to 2kb for the high-heterogeneity reads. The frequency at which this power spectrum peaked was 0.0335 corresponding to a wavelength of 187.6bp with the power being at least half of the maximum value at the peak for frequencies within

the range 0.0313–0.0357 (Fig. S5b,c). The spectral width therefore indicates that the oscillations have wavelength in the approximate range 176.0–200.7bp."

We have also included similar estimates of the wavelength along with other instances where we calculate distance dependent correlations.

We were not able to detect the previously reported 10bp periodicity from the power spectrum. We believe this could be because of the inability of Nanopolish to distinguish methylation states of CpG sites within 10bp of each other and have indicated this in our discussion (starting at line 567):

"In addition, Nanopolish is unable to distinguish methylation states between CpGs that lie in very close proximity of each other, leading to the same methylation state being called for CpGs within 10bp of each other. It is possible that this is why we do not observe the 10bp periodicity in the distance-dependent correlation previously observed from bulk bisulfite sequencing data that has been postulated to be representative of DNA winding around the DNA helix [27]."

We agree with the reviewer that it would be interesting to examine how distance-dependent correlations vary at each chromHMM state. However, to do so would require the development of a new analysis approach. Here we calculate distance-dependent correlations within whole single reads. To calculate correlations for each chromHMM annotation separately we would therefore be restricted to reads that lie entirely within each annotated state interval. The mean length of the reads used in our main analyses (i.e. those containing methylation information for at least 100 CpGs) is 41.5kb. This is 1-2 orders of magnitude longer than the mean length of the majority of ChromHMM state intervals (*Rebuttal Figure 1a*). In particular, the mean lengths of the intervals corresponding to the promoter, enhancer, insulator, transcribed region and polycomb-repressed states are 0.859kb, 0.860kb, 0.469kb, 3.84kb and 3.54kb respectively. The mean length of the heterochromatin state is more similar to the mean length of single reads (26.6kb). An analysis of distance-dependent correlations for the heterochromatin state reveals similar oscillatory patterns to those we report in low heterogeneity reads and in PMD reads (*Rebuttal Figure 1b*).

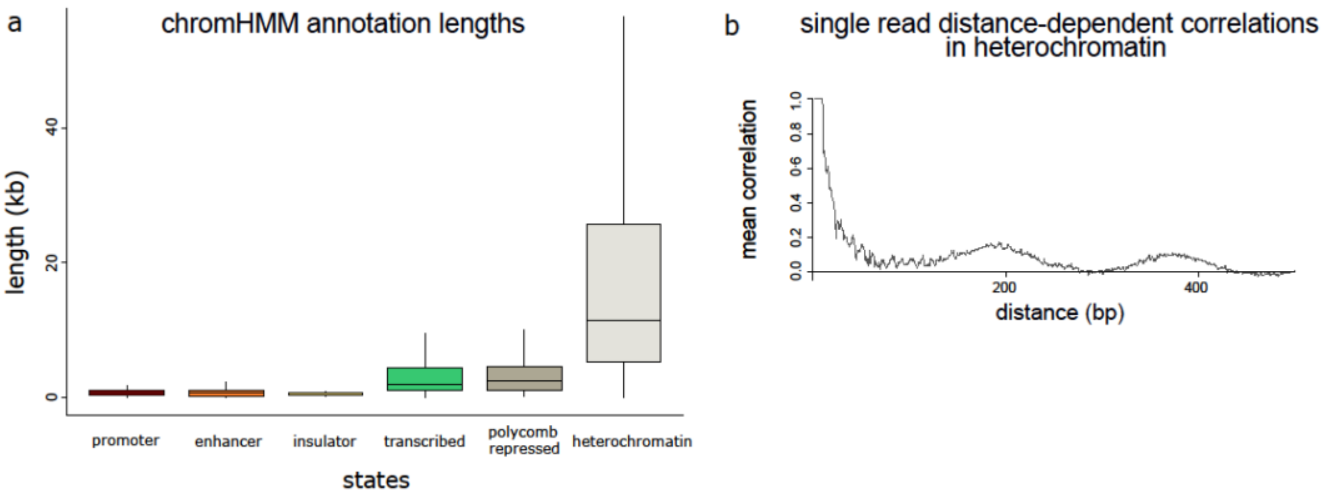

Rebuttal Figure 1: Oscillatory DNA methylation patterns are observed within single reads aligning to the ChromHMM heterochromatin state. a Box plot showing the distribution of lengths observed for intervals annotated with different chromHMM annotated states in GM12878 cells. Box = 25th–75th percentile; whiskers = 1.5 × interquartile range from box; n indicates the number of CpGs in each group. **b** Plot of mean distance-dependent correlation between CpG sites within 500bp of each other for 2,246,941 GM24385 reads aligning entirely within heterochromatic genomic regions.

4. typo in Fig5g title

We have now changed “synthetice” to “synthetic” in the title of Figure 5g.

5. some formatting issues in the bibliography (inconsistent capitalisation, ref57, etc)

We have rectified these issues in the revised manuscript.

Reviewer 2

Note, we have numbered Reviewer 2’s comments to facilitate cross-referencing.

Kerr et al. have presented a really careful and robust analysis of single-molecule of 5mC patterns in data from ONT whole genome sequencing of the GM24385 cell line. The manuscript is very clearly written and presented, with rationale for experimental design and choices being clearly explained throughout. The authors find intra-molecular 5mC heterogeneity, that was not possible to resolve from the analysis of bulk data alone. They find that this heterogeneity is largely enriched in ChromHMM annotated heterochromatin states, and further resolve this observation to show the greatest heterogeneity in previously described partially methylated domains. The authors observe an oscillatory pattern in single molecule 5mC distribution, and hypothesise that this could be the result of differential nucleosome phasing between molecules, and could underlie the observed 5mC heterogeneity. The authors observations are compelling, and I think that the following suggestions would enhance the manuscript:

We thank Reviewer 2 for their helpful suggestions that we feel have helped to improve the manuscript.

1. Can the authors analyse available NanoNOMe data to directly test their hypothesis that nucleosomes are the likely source of the intra-molecular 5mC heterogeneity observed in heterochromatic regions, by analysing 5mC and nucleosome occupancy on single molecules. These data have been generated in a different cellular context, but would be an important analysis nonetheless.

As suggested, we have incorporated an analysis of GM12878 nanoNOMe data into the revised manuscript. This analysis can be found in a new Figure 6 and starting at line 363.

Briefly, we calculated distance-dependent correlations in single reads between CpG sites, GpC sites and between the two types of site. We observe oscillations in CpG methylation within single high-heterogeneity GM12878 nanoNOMe reads replicating our original analysis. We also find that both high and low heterogeneity reads display GpC methylation oscillations. This suggests that the two groups having a broadly similar nucleosome organisation. By analysing the correlation between CpG and GpC methylation in single nanoNOMe reads, we find CpG methylation in high heterogeneity reads correlates with accessible linker DNA. Overall, this analysis reinforces the association between nucleosome positioning and DNA methylation within single molecules in heterochromatin.

2. Authors have analysed data from a single transformed cell line, GM24385. As authors have stated in their introduction, there are differences in both 5mC distribution and heterogeneity in cancer. How generalisable is this phenomenon? I would be interested to see whether the observed heterogeneity and oscillatory patterns in heterochromatin could be observed in a primary cells or pluripotent cells if the data is available, particularly because changes in partially methylated domains are hallmarks of cellular differentiation states.

We agree with the reviewer that it would be interesting to ask how DNA methylation patterns differ at the single-molecule level between primary and pluripotent cells or normal and cancer cells. However, we are not aware of any available Nanopore datasets that would allow us to address these

questions and the revision period was not sufficient to generate our own. We hope that our study will stimulate future work in this area.

In the revised manuscript, we provide analysis of data from GM12878 cells which replicates our key observations. In brief we show:

(i) That intra-molecular heterogeneity is non-randomly distributed in the GM12878 genome (Fig S2a,b, starting from line 136):

"To ensure that this variation in the degree of methylation heterogeneity across the genome was not specific to GM24385 cells, we also examined single-read correlations for the 1,327,908 GM12878 reads which had at least 100 CpGs. Both the distributions of read correlations and mean window correlations for GM12878 cells were bimodal (Fig. S2a,b)."

(ii) That regions of the genome with high intra-molecular methylation heterogeneity in GM12878 cells are enriched in heterochromatin and PMDs (Figs S4c and S4f, starting at lines 219 and 240):

"To ensure that these chromHMM enrichment results were not specific to GM24385 cells, we repeated the analysis for the least and most heterogeneous 100kb windows identified in GM12878 cells and observed analogous significant enrichment results as was observed in GM24385 cells (Fig. S4c)."

"To ensure that these PMD/non-PMD enrichment results were not specific to GM24385 cells, we also called 1,140 PMDs in GM12878 cells (designating the rest of the genome as 968 non-PMDs). We then repeated the analysis for the least and most heterogeneous windows in GM12878 cells finding the most heterogeneous windows to be significantly enriched in PMDs and the least heterogeneous windows to be significantly enriched in non-PMDs (Fig. S4f)."

(iii) That high-heterogeneity reads from GM12878 cells display oscillatory DNA methylation patterns (starting at line 296):

"To ensure that this periodicity was not a feature specific to GM24385 cells, we repeated the analysis using the data from GM12878 cells. GM12878 high heterogeneity reads displayed oscillations that were similar to those seen in GM24385 cells and that were largely missing in low heterogeneity GM12878 reads (Fig. S5a)."

Minor comments:

3. Authors state read count and mean read length of the data analysed. Could they also add the genome coverage obtained in the data analysed.

We have now added information regarding CpG coverage for the datasets analysed (starting at line 45):

"The mean coverage of each autosomal reference CpG was ~39.1. We validated certain key results using Nanopore GM12878 data, [19] (see Methods) which consisted of 10,784,132 reads aligned to autosomes with a mean length of 8.7kb and with mean autosomal reference CpG coverage of 18.6."

4. When authors were comparing inter- and intra-molecular heterogeneity, they looked at di values for the highest and lowest correlating 10% 100kb windows. I'd like to see what this correlation looks like genome wide, in the more intermediate windows too.

We now include an analysis of *di* values in all 100kb genomic windows. We find that mean window *di* correlates positively with mean window correlation, reinforcing the conclusion that intra-molecular and inter-molecular methylation heterogeneity co-occur in the human genome (Figure S3b and starting at line 184):

"In order to examine how inter-read heterogeneity and intra-read heterogeneity correlated genome-wide, we then examined the association between mean di value and mean correlation in all 100kb genomic windows. In agreement with high intra-molecular heterogeneity coinciding with high inter-molecular heterogeneity, we observed a significant positive correlation between mean di and mean correlation (Fig.S3b, Spearman correlation = 0.632, $p<2.20 \times 10^{-16}$)."

Reviewer 3

Note, we have numbered Reviewer 3's specific comments to facilitate cross-referencing.

In this submission, the authors present a series of computation analyses on Nanopore sequencing data from a single individual that leads them to conclude methylation heterogeneity is common in heterochromatic regions of the genome, and that such heterogeneity appears to follow some periodic pattern that resemble those that nucleosomes appear to cause.

The questions that the authors address are interesting and I am convinced their analyses and the conclusions they reach from them are interesting for the research community, but, in my opinion, the current version of the manuscript still requires work to be ready for publication. I hope that my comments below will help the authors achieve that goal.

We thank the reviewer for acknowledging the interest of our work and for their comments and suggestions that we feel have strengthened our results.

1. Replication.
All analyses are conducted on data from a single individual. Why didn't the authors use the publicly available Nanopore sequencing data for GM12878, from which the chromHMM-defined regions are obtained?

As detailed in response to reviewer 2 (point 2), the revised manuscript contains an analysis of GM12878 data which replicates our key observations.

2. Differences between bulk and per-read analysis.
The first result presented in the manuscript is that heterogeneity estimates from single-read data are higher than those obtained from bulk values. I am not sure, however, if the conclusion that the authors reach from this observation is warranted. According to them, this indicates that bulk values are "underestimates", but, as far as I understand it, there is no reason to believe that the per-read analysis leads to the actual ground truth value. It is possible that the bulk values correct for the potential methylation call errors that the per-read estimates cannot, and thus they are closer to the real values. Unless some other analysis supports that the difference between the two methods?

We acknowledge that there will be errors in the single-molecule methylation data. However, this will also be true for the bulk data which are directly calculated from the single-molecule methylation calls. To reduce errors in the single-molecule data we filter out low-confidence methylation calls. We also only consider single-read patterns consisting of a minimum of 100 called CpGs. This means that the impact of errors introduced by a small number of miscalled CpGs in a read on the statistics calculated for that read is likely to be low. In the revised manuscript, we include discussion of the impact of methylation calling errors on our analysis (starting at line 552):

"To deal with erroneous methylation calls within single molecules, we filter out low-confidence Nanopolish methylation calls, though we note that such filtering may bias against certain motifs and sequence contexts. We also restrict our attention to single-molecule patterns consisting of a minimum of 100 called CpGs, meaning the impact of a small number of miscalled CpGs when calculating read statistics should be minimal."

3. Causes for the observations.
The authors put a great deal of effort into attempting to explain the phenomena that they report. I am not sure, however, whether the analyses that they conduct allow them to conclude much regarding causality.

The reviewer is correct that our study does not prove that nucleosomes cause the oscillatory methylation patterns we observe. We have included discussion of this point and alternative possibilities in the revised manuscript (starting at line 516):

"We note that our analyses examine the correlation between accessibility and DNA methylation and cannot determine causal relationship between DNA methylation and nucleosomes. It is possible instead that the patterns arise due to the influence of DNA methylation on nucleosome positions. For example, DNA methylation has been reported to affect the interaction of nucleosomes with a positioning sequence in the chicken beta-globin locus [49]. In addition, although nucleosome positioning is thought to the major global influence on accessibility measured by CviPI methylation [29], other proteins can also affect accessibility, including transcription factors [50]. Further work will be required to precisely dissect the nature of the relationship between nucleosome occupancy and DNA methylation."

4. Bulk methylation.
Between lines 280 and 296, the authors address whether the patterns they observe originate from the bulk methylation levels by generating synthetic reads in which the probability of each site to be methylated depended solely on the bulk methylation level at that site. As far as I understand it, based on visual inspection of Figure 5d, the oscillation patterns detected on such synthetic data match very closely those in Figure 5a except perhaps at very close distances. The frequency of the oscillation, in particular, appears to be spot on, and that is, in my opinion, the most relevant observation. In addition, the difference in close distances can be easily explained if one considers that contiguous or close to contiguous sites (such as in CpG islands) are much more likely to be highly linked than randomly, but at distances where that coupling would be less likely the patterns appear to match. I think it is not warranted that the authors claim the patterns "differ". It may well be that bulk methylation alone cannot explain the entirety of the pattern (since there are certainly non-random procedures at work), but that does not mean it is not a cause, even if only partially.

In the revised manuscript, we have statistically compared the distance-dependent correlations from the real high heterogeneity and the synthetic high heterogeneity reads showing that they are significantly different. We have also added a statement indicating that bulk methylation patterns partially explain the patterns observed in single-molecules (starting at line 328):

"The mean distance-dependent correlations derived from synthetic high heterogeneity reads showed oscillatory behaviour with wavelength in the range 177.5-198.3bp but the magnitude of these oscillations was significantly lower than that observed from the real high heterogeneity reads (Fig. 5d, Wilcoxon ranked sign test, p=0.003). This suggests that bulk methylation levels only partially explain the patterns observed in single molecules."

5. Nucleosomes.
(i) The alternative explanation that the authors attempt to test is whether the patterns could be derived from nucleosome organisation. In fact, that is the explanation that the authors appear to favour, as the conclusions clearly state: "We propose that the nature of this heterogeneity suggests a role for nucleosomes in shaping DNA methylation patterns at the single-molecule level, particularly in heterochromatin". For that reason, it feels strange that all plots leading to this prominent conclusion are placed exclusively on the supplementary information instead of as part of the main figures 5 or 6. If the results are so important that they lead to one of the main conclusions of the manuscript, they should be featured accordingly, which would also help their scrutiny.

As detailed in response to reviewer 2 (point 1), the revised manuscript includes an analysis of nanoNOMe-seq data which reinforces the conclusion that the oscillatory patterns in single molecules correlate with nucleosome organisation. The results of these analyses are included in the main Figure 6.

(ii) Although not explicitly stated in the conclusions, the authors mention at the end of the results section that the "patterns differ from those predicted from mean bulk DNA methylation and could result from differences in nucleosome phasing between molecules" because they consider the recurrence plots generated from the artificial "unphased nucleosome" reads to be "in qualitative agreement" with those from the real reads. Although it is difficult to compare "qualitatively" (and specially since the plots are not presented closely), I would personally say that Figure 5f is more "qualitatively similar" to 5e than S5h is to 5f.

In order to facilitate comparison of the results of data analysis and modelling, we now explicitly state and label the features which are in qualitative agreement between the recurrence plots generated from the models (Fig S6) and experimental data (Fig 5) (e.g. starting at line 447):

"We next generated data from a model where nucleosome positioning was completely unphased between molecules (Fig S6d, see Methods). We again observed large, regular blue areas corresponding to regions of high similarity in the correlations associated with different distances x and y (Fig. S6e arrows 1 and 2) which had distinct boundaries from yellow elliptical regions corresponding to regions of low similarity (Fig. S6e arrow 3). However, the recurrence plot generated using the bulk properties of the model showed correlations to be more similar across distances in comparison to the single-molecule recurrence plot generated from the model (Fig. S6e,f). The blue areas indicated by arrows 1 and 2 in Fig. S6e,f are larger in the recurrence plot associated with the synthetic reads in comparison to the recurrence plot associated with the real reads. This results in near complete occlusion of the yellow elliptical region indicated by arrow 3 in the recurrence plot associated with bulk properties of the model. Our observations from this model qualitatively agree with the differences we observed in the recurrence plots generated from real and bulk-simulated high-heterogeneity reads (Fig 5e—g arrow 3), consistent with the hypothesis that these differences could be generated by differences in nucleosomal organisation between molecules."

(iii) Aside from that, the analysis itself confuses me. I fail to see how this largely qualitative analysis says much about the role of nucleosomes themselves. If I understood it correctly, the analysis involves artificially creating periodic (or pseudo-periodic) patterns of methylation, and see if those somehow match patterns that have already been shown to be periodic. Whether those pseudo-periodic patterns arise from nucleosomes or something else does not seem to really play a role here.

The reviewer is correct that proteins other than nucleosomes could potentially cause differences in the ability of DNMTs to methylate DNA and give rise to the oscillatory patterns we observe. In addition, although the major determinant of accessibility measured by CviPI methylation is thought to be nucleosomes, they are not the only determinant of GpC methylation in this assay. We have added discussion of this point to the manuscript (starting at line 521):

"In addition, although nucleosome positioning is thought to be the major global influence on accessibility measured by CviPI methylation [29], other proteins can also affect accessibility, including transcription factors [50]. Further work will be required to precisely dissect the nature of the relationship between nucleosome occupancy and DNA methylation."

(iv) The creation of the "phased" reads also confuses me. If all reads have the "nucleosomes" placed in the exact same positions and the methylation depends exclusively on where those "nucleosomes" fall, are these reads not all exactly the same one? And is it really worth it to check for "bulk" patterns

in this case if all reads will match exactly? What is the difference between a single read and multiple that have the exact same methylation state at each position?

To address this point, we have assigned methylation state in our models in a probabilistic manner dependent on nucleosome occupancy in the revised manuscript. In particular, nucleosome-occupied DNA is unmethylated with probability p=0.8 and unoccupied DNA is methylated with probability p=0.8. The probabilistic element introduced here means that while nucleosome occupancy is identical between the modelled phased reads, they have variable methylation states. We have detailed this in the results (starting at line 431):

"…methylation state was determined in a probabilistic manner based on nucleosome occupancy."

and the methods (starting at line 859):

"As nucleosomes are refractory to the action of DNMTs, we assumed that each nucleosome occupied DNA base was unmethylated with probability p=0.8 and each linker DNA base was methylated with probability p=0.8 [38-42]."

(starting at line 878):

"However, we note that while each read in this model was associated with the same nucleosome occupancy pattern as R_1, the associated methylation pattern varied from read to read due to the probabilistic way in which methylation state was assigned to occupied and unoccupied DNA bases."

(v) Related to the creation of those reads, does the statement in Line 656 that "we assumed that every DNA base on the molecules could be methylated" imply that literally any base, no matter its nucleotide, was considered methylated in the linker sequences between the "nucleosomes"? If that obviously unrealistic assumption is indeed made, I would appreciate it if there was some validation of its implications.

Our aim was to use simple models to gain intuition as to the potential causes of the differences we observed in single molecule methylation patterns compared to what is expected from bulk. As such we did not seek to accurately capture every aspect of the system in detail. If DNA methylation patterns were strongly dictated by nucleosome occupancy, then methylation patterns would reflect a sampling of nucleosome positioning at genomic positions where CpG sites are present. In the models we sought to reduce the number of reads we had to simulate by increasing the frequency of sampling through considering the case that every base could be methylated. We have provided additional explanation of this in the revised manuscript (starting at line 855):

"If methylation state was strongly dictated by nucleosome occupancy, then DNA methylation data would sample of nucleosome occupancy data where CpG sites are present. We therefore simplified our models and made them more tractable with a lower number of simulated reads by assuming that every DNA base on the molecules could be methylated."

(vi) In addition, in the discussion (lines 408 to 411) the authors state that sequence influences nucleosome positioning. If that is the case, would that not imply that nucleosomes would tend to be phased (i.e., located in similar loci on multiple molecules, since those loci would contain similar, if not identical, sequence)?

The reviewer is correct that if sequence determined nucleosome positions, then they would be predicted to be phased across molecules. However, nucleosome positioning sequences from the mouse genome has previously been reported to have affinities that are 6-fold lower than those observed for optimised synthesised sequences such as the Widom 601 sequence (Thastrom et al 1999 Journal of Molecular Biology, DOI: 10.1006/jmbi.1999.2686). In addition, many experimental studies indicate that the majority of nucleosomes are not phased in human cells (discussed in Baldi et al 2020 Nature Structural and Molecular Biology, DOI: 10.1038/s41594-019-0368-x). This suggests

that while sequence influences nucleosome positioning it does so non-deterministically in cells. We include discussion of the lack of phasing (starting at line 487) and influence of sequence on nucleosome positioning (starting at line 533) in the manuscript:

"...only an estimated 8.7% of nucleosomes in the human genome are strongly-positioned [44] and the majority are not phased between molecules [35]. A single-molecule analysis of Micrococcal nuclease digested fragments suggests that heterochromatin is characterised by regularly spaced nucleosomal arrays [45]. Regular nucleosome spacing in heterochromatin was also observed in a single-cell nucleosomal positioning study which additionally suggested that heterochromatin is characterised by a lack of phasing between molecules [46]. In addition, a single-molecule footprinting analysis of oligonucleosomes released from cells found that irregular oligonucleosome patterns were abundant in heterochromatin [47]."

"However, nucleosome positioning is also non-deterministically influenced by sequence [55]…"

6. CpG density.
I feel that the analysis reported here largely fail to account for the effect of CpG density on the methylation state of particular regions. For instance, when comparing heterogeneity values between heterochromatic and euchromatic regions, it would make sense to do so on equivalently dense windows, since CpG islands, for example, will affect the results. Also, the specific heterogeneity metrics, such as the "correlation" or RTS do not take into account how far away the "neighbouring" sites are, and one would expect that contiguous sites are more likely to be in sync than those that are potentially thousands of bases away (on 100 kb windows, a minimum threshold of 100 CpG sites can lead to very distant "neighbours").

We agree that CpG density is an important factor to consider. However, it is not possible to robustly dissect its role relative to nucleosomes and DNA sequence within the time frame of the revisions due to mutual correlations between all these factors. We have added discussion of previous studies regarding the role of CpG density in reinforcing homogenous methylation patterns to the revised manuscript (starting at line 535):

"In addition, both modelling and experimental studies suggest that collaborative interactions between proximal CpG sites are required to maintain DNA methylation patterns [57-59], suggesting that local CpG density could also modulate DNA methylation patterns by reinforcing homogeneity in CpG dense regions. Collaborative methylation interactions are likely determined by the properties of the DNA methylation machinery. DNMT1 and DNMT3B can both methylate processively along DNA strands and DNMT3A forms multimers along the DNA fibre [60]. It is therefore possible that any associations existing between nucleosome positioning and DNA methylation could be linked with DNA sequence and CpG density, and future studies will be required to precisely dissect their respective roles.
